# Regulation of the Demographic Structure in Isomorphic Biphasic Life Cycles at the Spatial Fine Scale

**DOI:** 10.1371/journal.pone.0092602

**Published:** 2014-03-21

**Authors:** Vasco Manuel Nobre de Carvalho da Silva Vieira, Marcos Duarte Mateus

**Affiliations:** MARETEC, Instituto Superior Técnico, Universidade Técnica de Lisboa, Lisboa, Portugal; Dauphin Island Sea Lab, United States of America

## Abstract

Isomorphic biphasic algal life cycles often occur in the environment at ploidy abundance ratios (Haploid:Diploid) different from 1. Its spatial variability occurs within populations related to intertidal height and hydrodynamic stress, possibly reflecting the niche partitioning driven by their diverging adaptation to the environment argued necessary for their prevalence (evolutionary stability). Demographic models based in matrix algebra were developed to investigate which vital rates may efficiently generate an H:D variability at a fine spatial resolution. It was also taken into account time variation and type of life strategy. Ploidy dissimilarities in fecundity rates set an H:D spatial structure miss-fitting the ploidy fitness ratio. The same happened with ploidy dissimilarities in ramet growth whenever reproductive output dominated the population demography. Only through ploidy dissimilarities in looping rates (stasis, breakage and clonal growth) did the life cycle respond to a spatially heterogeneous environment efficiently creating a niche partition. Marginal locations were more sensitive than central locations. Related results have been obtained experimentally and numerically for widely different life cycles from the plant and animal kingdoms. Spore dispersal smoothed the effects of ploidy dissimilarities in fertility and enhanced the effects of ploidy dissimilarities looping rates. Ploidy dissimilarities in spore dispersal could also create the necessary niche partition, both over the space and time dimensions, even in spatial homogeneous environments and without the need for conditional differentiation of the ramets. Fine scale spatial variability may be the key for the prevalence of isomorphic biphasic life cycles, which has been neglected so far.

## Introduction

Algae species with isomorphic biphasic life cycles have their alternating haploid and diploid generations cohabiting. The ratio between the field abundances of these opposite ploidy phases (H:D, or G:T for Gametophyte:Tetrasporophyte) has long been an intriguing subject. It would be expected to find balanced abundances between ploidy phases as a consequence of isomorphicity. However, these often are uneven as reported in several studies [1]–[7]. The persistency of uneven ploidy phase abundances may be taken as evidence of niche partitioning. Hughes and Otto [8] have mathematically proved the necessity for a niche partition for one of the ploidy phases to not exclude the other, eliminating the biphasic life cycle and fixing it as a monophasic one. In their non-spatial model solved for steady-state, the niche partition was due to conditional differentiation of the ploidy phases and led to a fixed H:D.

Conditional differentiation means separate entities differentiating the way they adapt to the environment in order to coexist. Haploids and diploids of isomorphic biphasic life cycles have shown to possess subtle morphological differences [9], divergent metabolic rates [10] and distinct biochemical constitutions [11], besides their well established cytological differences in spore production (i.e. meiosis *vs* syngamy). These characteristics affect the individual vital rates [11]–[16], which in algae are commonly classified as fecundity, spore survival, ramet growth and ramet survival. Furthermore, when a species focuses its energy budget and demography on a type of vital rate it is said to have its life strategy dominated by that type. Conditional differentiation implies that if one ploidy is best adapted to some circumstances, the other is best adapted to others. Gonzalez and Meneses [13] observed haploids of the red algae *Chondracanthus chamissoi* perform better at spore settlement and germination whereas the diploids perform better at drifting spore survival, ramet growth and fecundity. Pacheco-Ruíz *et al*. [16] observed *Chondracanthus squarrulosus* released much more carpospores than tetraspores but the latter had much higher germination rates. Furthermore, the diploid ramets responded negatively to high temperatures whereas the haploid ramets did not.

For any particular location the environment changes seasonally. Thus, it is expected for the two ploidy phases that conditionally differentiate to seasonally shift their field dominance, as observed by Dyck and DeWreede [7], Engel *et al*. [2], Otaiza *et al.* [17] and Thornber and Gaines [3]. Nevertheless, using a theoretical modeling approach, Vieira and Santos [18] have determined cyclic alternations in phase dominance may arise as a sole consequence of a ploidy asymmetrical life cycle structure with fixed ploidy differences in features like size of first reproduction or maximum frond size. Habitats also change widely with geographical location and the H:D of a particular species changes accordingly [3], [7]. In another theoretical modeling study Vieira and Santos [19] have demonstrated the large geographical variation of the H:D observed by Engel *et al*. [2] and Thornber and Gaines [3] could easily be generated by ploidy dissimilar looping (stasis, breakage and clonal growth) rates in species with looping dominated life strategies, whereas it could hardly be generated by ploidy dissimilar fertility rates. Yet, the spatial variability of the H:D does not occur only at a large geographical scale between populations clearly set apart and subject to distinct environments. It has also been documented within the same population related to intertidal height, degree of hydrodynamic stress and distance from shore [2], [4], [6], [7], [12], [20]. It was the objective of this work to determine which ploidy differences may induce (more or less efficiently) the required niche partition at a fine spatial resolution (i.e. intra-population). Modeling population dynamics with simulated data were used to investigate the effects of ploidy dissimilarities in growth and looping of the ramets, in fecundity and in spore dispersal. The last two do not necessarily result from conditional differentiation but rather from the differences in haploid and diploid cytologies [21]–[23].

## Methods

**Figure 1:**
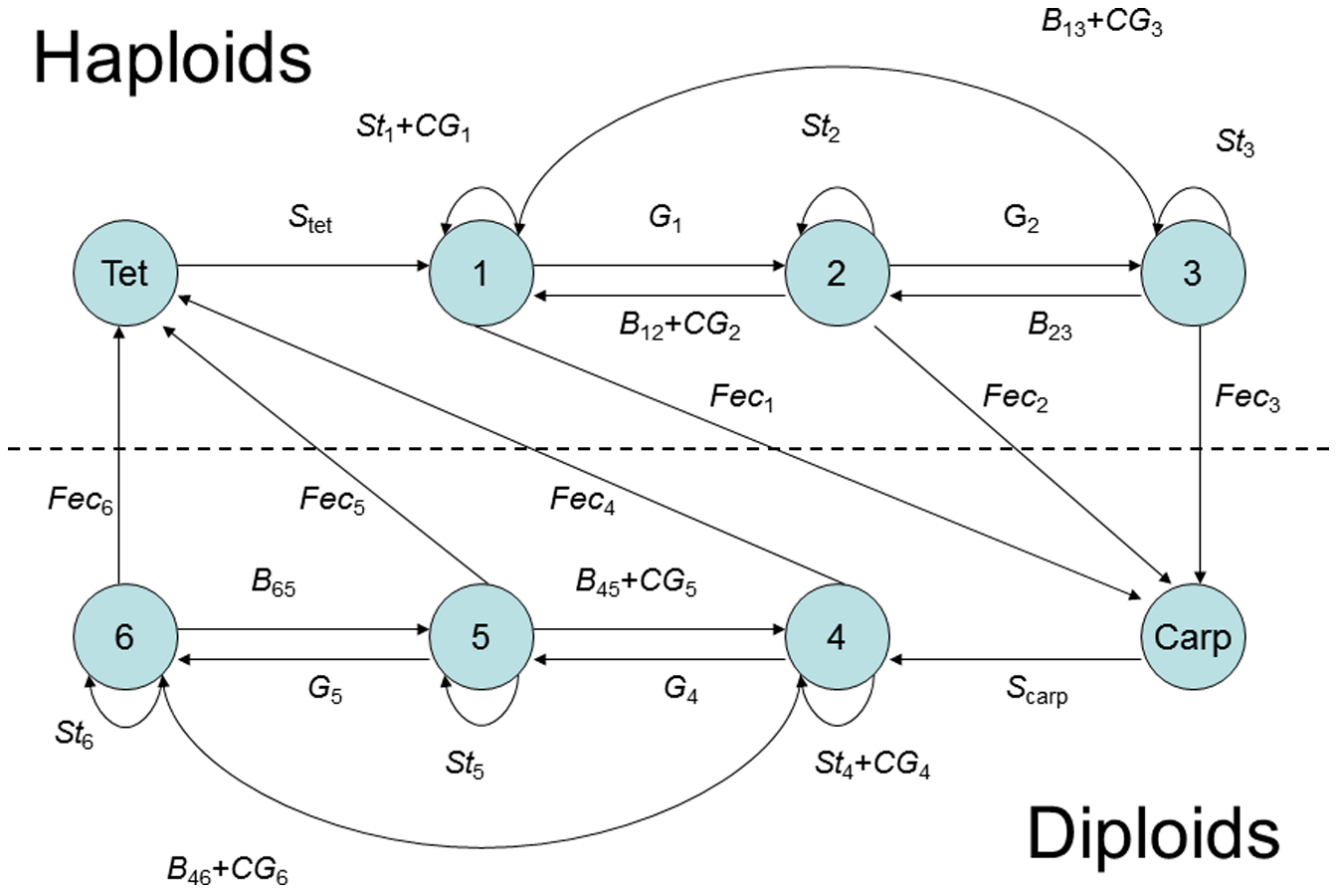
Biphasic life cycle. (Tet) tetraspores, (1, 2 and 3) gametophytic ramet size classes, (Carp) carpospores, (4, 5 and 6) tetrasporophytic ramet size classes, (cg) clonal growth, (fec) fecundity, (s_tet_) tetraspore survival, (s_carp_) carpospore survival, (g) growth, (st) stasis and (b) breakage.

The non-spatial biphasic life-cycle model was adapted from Vieira and Santos [24], where the projection interval (Δ*t*) is one month and each phase has one spore stage and three ramet size classes (Fig. 1). This way it was possible to consider biological processes as ramet growth, fecundity allometry and spore dynamics. However, the demographic matrix was generalized for parsimony and not adjusted to any particular population or species (equation 1): (i) the haploid and diploid fecundities (fec) were dependent on the parameters *f_H_* and *f_D_* and a size class weight factor (*wf_j_*, *j* is the column index) accounting for allometry; (ii) the survival of the carpospores (*S_carp_*) and of the tetraspores (*S_tet_*) was kept constant; (iii) the haploid and diploid ramet growth rates were dependent on the parameters *g_H_* and *g_D_*; (iv) the haploid and diploid stasis, breakage and clonal growth were aggregated as looping rates and dependent on the parameters *l_H_* and *l_D_*. It was named “looping” for consistency with previous works by Vieira and Santos [18], [19], [24] and is equivalent to retrogression plus stasis [25], [26], [27]. All ramet stages of the same ploidy had the same amount of looping. Therefore, *l* was partitioned along each column so that its sum was *l_H_* or *l_D_*. Furthermore, the column sums of *l* and *g* (i.e. survival) could not exceed 1. Table 1 shows the notation used for the model parameters and analysis.

**Table 1:** Notation used for model parameters and analysis.

symbol	parameter	symbol	parameter
H	haploid	Fv	fertility vector
D	diploid	N	Population vector
f	fecundity	T	Ramet transition matrix
wf	size class weight factor for f	I	identity matrix
sw	spore survival while suspended	t_a_	advanced time at steady-state
ss	spore survival while settled	x	x spatial dimension
dr	diffusive radius	a	x begining
g	ramet growth	b	x end
l	ramet looping	y	y spatial dimension
F	fertility life strategy	c	y beginning
G	growth life strategy	d	y end
L	looping life strategy	disx	ploidy diss. coef. along x
λ	population growth rate	disy	ploidy diss. coef. along y



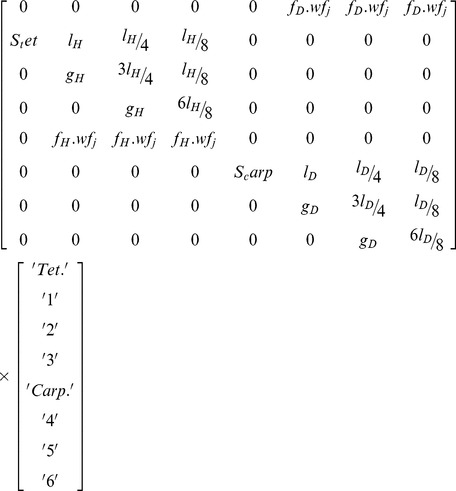
(eqn1)


**Figure 2:**
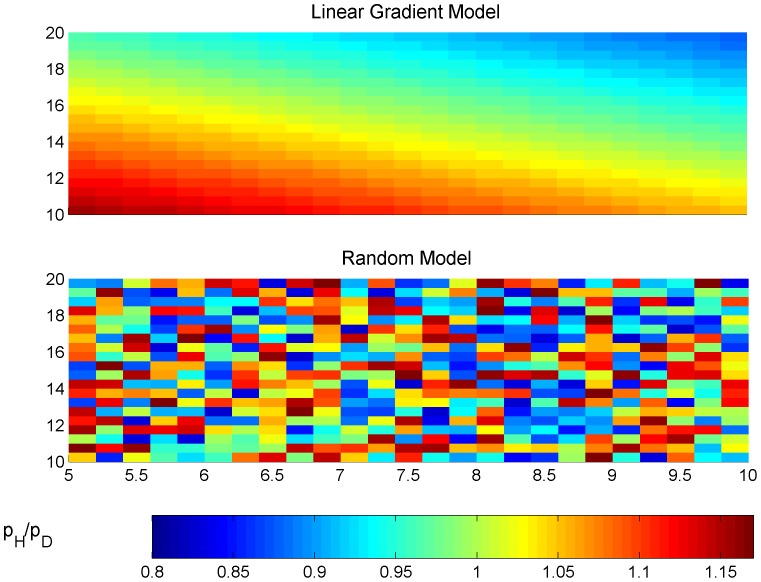
Spatial distribution of phase dominance in parameter *p*. (upper panel) given by the linear gradient model with *disx*  = 0.05 and *disy*  = 0.1, and (lower panel) given by the random model with *disx*  = 0.25 and *disy*  = 0.25. Grid defined by *a* = 5, *b* = 10, *dx* = 0.2, *c* = 10, *d* = 20, *dy* = 0.5; Parameter *p* = 0.35.

Spatial discretization was inserted assuming a population scattered over a grid defined by two orthogonal axes. The *x* dimension was evaluated from point ‘*a*’ to ‘*b*’ at intervals of Δ*x* whereas the *y* dimension was evaluated from point ‘*c*’ to ‘*d*’ at intervals of Δ*y*. These axis are scalars, meaning that they are unitless. The fundamental aspect is that space discretization is unitless throughout this work, thus providing the possibility to choose the most suited units for each particular case. Typically these units are within meters to decameters. A demographic matrix was estimated for each point with its specific ploidy dissimilarities for fecundity (*f*), growth (*g*) and looping (*l*) rates. These dissimilarities were tested according to two different hypotheses:

1) For each type of vital rates at a time there was a dissimilarity linear gradient benefiting the haploids in ‘*a*’ and ‘*c*’ and the diploids in ‘*b*’ and ‘*d*’ (Fig. 2, upper panel). This scenario may represent a cross shore gradient of intertidal height (usually occurring over a few tens of meters) and an along shore gradient of wave exposure or fresh water influence (usually occurring over tens to hundreds of meters), as described for isomorphic biphasic life cycle populations [2], [4], [6], [7], [12], [28]. The levels of ploidy dissimilarity, named ‘*disx*’ for the *x* dimension and ‘*disy*’ for the *y* dimension, were defined as the surplus in the parameter of one phase relative to the opposite phase. Therefore, for any point (*x*,*y*), the parameters had their values estimated for the haploids and diploids as in Table 2.

**Table 2:** Estimation of the parameters *p_H_* and *p_D_* in point (*x*,*y*) according to dissimilarity levels *disx* and *disy*.

Phase	Parameter *p* at point (*x*,*y*):	
	linear gradient model (hypothesis 1)	random model (hypothesis 2)
H	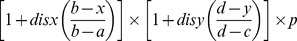	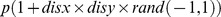
D	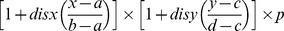	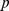

2) The ploidy phase dissimilarities in fecundity, growth and looping rates were randomly distributed over space (Fig. 2, lower panel). For any point (*x*,*y*) inside the grid the haploid and diploid vital rates were estimated as in Table 2. The maximum surplus was given by *disx* × *disy*. This scenario simulates, for example, a rocky intertidal area with rock pools (usuallya few meters wide maximum) at different elevations where the environment inside the pools is different among them and from the environment outside. This situation is documented for the isomorphic biphasic life cycle species *Gracilaria verrucosa* [12].

At this stage the model was unable to simulate dispersal along the grid. This was established assuming only the spores dispersed by diffusion. Advective transport was neglected for simplicity. Consequently, the biological resolution of the life cycle was increased following Destombe et al [10]: during one time step the spores were released to the water column, dispersed, subject to a survival rate (*sw*) and finally settled. At the end of the projection interval the surviving spores made the transition to either the carpospore or tetraspore stage (equation 2) whereas the remaining either died or exited the population (which was demographically equivalent to having died):
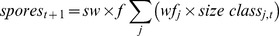
(eqn2)


It was assumed that spores had a diffusive radius (*dr*) corresponding to the maximum distance they could travel away from their release point while drifting. This was dependent on the time spores spend drifting and on the spore sinking rate, which differs with ploidy. Therefore, also the diffusive radius could differ with ploidy. The spores were assumed to be randomly spread within the circumference defined by the release point and diffusive radius. Thus, the probability of one spore released from point (*x*_0_,*y*_0_) to arrive at point (*x_p_*,*y_p_*) could be approximated by Δ*x*Δ*y*/(π*dr^2^*). The spores at point (*x*_0_,*y*_0_) and time *t+*1 could then be estimated by equation 3. For the discrete model implementation the integral was estimated numerically as Riemann's integral. In the following time interval the settled spores germinated and grew to the first size class subjected to a survival rate (*ss*) corresponding to *S_carp_* or *S_tet_* depending on the ploidy phase.
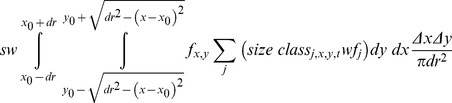
(eqn3)


The simulation of spore dispersal was incorporated into the demographic model by splitting the demographic matrix into its transition and fertility components. This was done in a slightly different way from what is usually done in Markov chain analysis. The demographic model (equation 4) became **N_t+1_** = **T**×**N_t_**+**Fv_t_** where **T** is the square matrix of transitions of individuals older than one projection interval and **Fv** is the fertility column vector containing spore production and dispersal. To estimate **Fv** eqs 2 and 3 where applied separately to haploids and diploids.
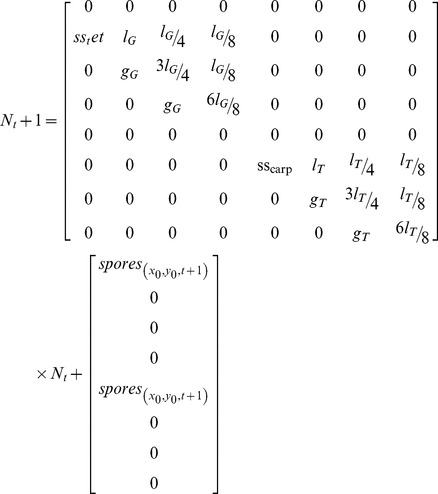
(eqn4)


The demographic model was tested for a wide range of fertility, growth and survival rates intending to generate a set of demographic matrices covering the broadest possible combinations fitting real world situations. Growth (*g*) and looping (*l*) were randomly selected from 0 to 1 constrained to survival (the column sum of *g* and *l*) could not exceed 1. Spore survival (the product *sw* × *ss*) was randomly selected from 10^−6^ to 10^−2^. Following this procedure were generated 10000 demographic matrices but only accepted those where the asymptotic growth rate (λ) was between 1 and 1.1. Below 1 the population faces extinction whereas at 1.1 (corresponding to a 3.14 yearly growth rate) it is a demographic burst.

The population growth rate (λ) does not have an analytical solution when at least some model parameters do not have fixed values. In the present case the entries in the F vector were dependent on the abundances of ramets in neighbor locations. Therefore, λ needed be estimated numerically. Being the population growth a multiplicative process, λ was estimated from a geometric formula and not arithmetic, given by λ = (*N_ta+t_*/*N_ta_*)^1/*t*^; where *t_a_* is an advanced time where the population is already at steady state. A life strategy dominated by fertility (F), growth (G) or looping (L) was numerically determined as one where the bulk of the elasticities of λ were to the *f*, *g* or *l* entries in the demographic matrix. This type of classification was introduced in plant demography by Franco and Silvertown [29] and Oostermeijer *et al.* [25]. It was later applied to the demography of isomorphic biphasic life cycles in Vieira and Santos [18], [19], [24].

The demographic model dynamics were semi-analytically determined for steady-state (i.e. analytical solutions for most demographic aspects still depended from numerical estimates of λ) and numerically determined for its preceding transient phase. Steady-state was always possible as the populations always tended asymptotically to an ergodic (i.e. independent of the initial conditions) stable structure (**N**) and growth rate (λ): the spatially explicit population model could be alternatively written as one single demographic matrix with a main diagonal of blocks (with a specific **T** for each location in each block) whereas the upper and lower triangles would have the blocks with the specific spore migration between pair-wise locations. This matrix is irreducible and primitive according to the Perron-Frobenius theorem, having a dominant eigenvalue which sets the asymptotic growth rate and an associated eigenvector which sets the asymptotic population structure [30]. Following the model presented in equation 4 at any point (*x*_0_,*y*_0_) inside the population grid it was possible to determine a semi-analytical solution to the stable population structure (equation 5):
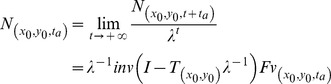
(eqn5)where the **Fv** and **N** vectors are standardized to the population size at time *t_a_* and **I** is the identity matrix. Eqn. 5 has affinity with the Lotka's integral equation for the instantaneous growth rate *r*, Volterra's integral equations and the McKendrick-von Förster models. Its deduction is available in Appendix S1. The maternity or birth functions integrate **Fv** and include immigrant spores.

There are many estimates of vital rates from natural populations and/or artificial cultures. Some of these works focus on particular rates ignoring the simulation of a full life cycle [12], [13], [14], [16]. Others allow a full cycle simulation [2], [22] but relate to largely different model structures disabling any honest fit to the present model. Incompatibilities include (i) one year projection intervals, (ii) haploids split into males and females, and (iii) absence of size classes turning ramet growth rates undetermined. The model was tested with the *Gelidium sesquipedale*'s vital rates previously estimated by Santos and Duarte [9] and Santos and Nyman [1] and summarized in Vieira and Santos [24]. These neglected differences between ploidies when estimating the growth and looping rates. However, Carmona and Santos [10] found growth of re-attached diploid fronds to be about 1.2 times that of haploid fronds. The *G. sesquipedale* matrix T was adapted accordingly (equation 6). Carmona and Santos [10] also found reproductive structures releasing carpospores for twice as much time as tetraspores. This was consistent with the previous works estimating haploid fertility about twice as high. Thus, the model was tested with *f* = 7000 and **wf** = [0 0 2 4 0 0 1 2]. Furthermore, Carmona and Santos [10] found temperature dependent ploidy dissimilarities in spore attachment and germination, benefiting opposite ploidies in winter and summer extremes. At 13°C carpospores attached to the substrate about twice as better as the tetraspores, whereas at 21°C it was the opposite situation. Although this represents a seasonal variability it was decided to convert this data to a spatial variability and test it as merely an indicative of the potential effects of such ploidy dissimilarities. This was done post-multiplying the carpospores and tetraspores in the **F** vector by a 0.5 attachment rate and simulating its spatial evolution accordingly to the linear gradient model (in Table 2) with *disx* = 1.
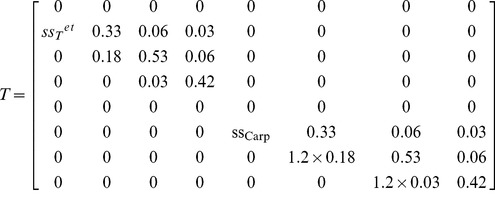
(eqn6)


## Results

### Effects of ploidy dissimilarities in fertility, growth and looping without spore dispersal

**Figure 3:**
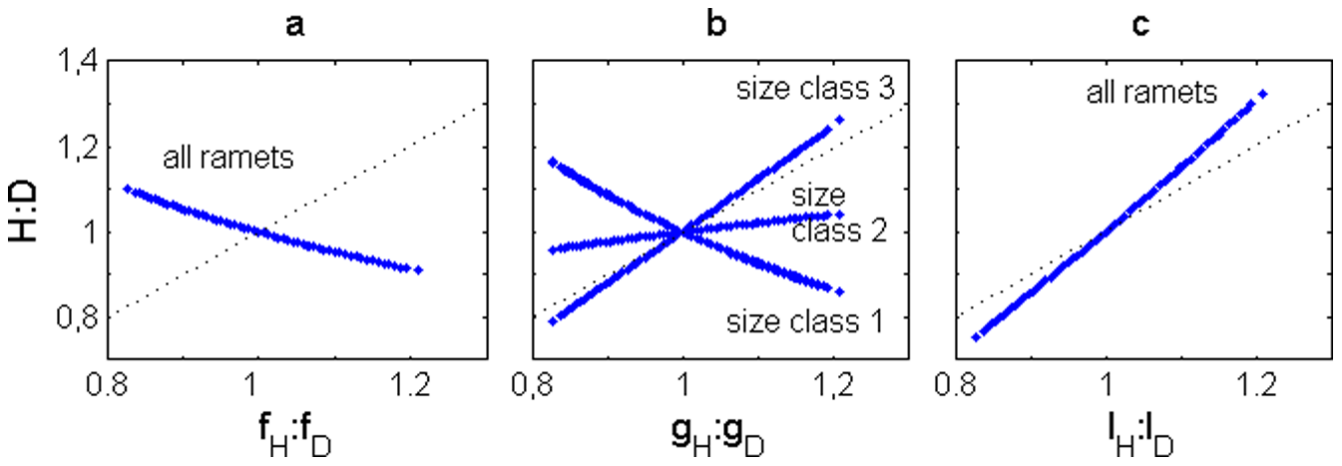
H:D under no dispersal. In (a) fertility, (b) growth and (c) looping life strategies. *x* axis: the respective vital rates ratio in each point. *y* axis: assymptotic H:D in each point. Dotted line: *y* = *x* bisect.

The absence of dispersal was simulated setting *dr_tet_* = *dr_carp_* = (Δx.Δy/π)^1/2^ = 0.5642 so that the fertilities given by equations 2 and 3 were not influenced. The steady-state H:D followed the patterns set by the linear gradient model (Fig. 2 upper panel) and the random model (Fig. 2 lower panel). However, these patterns were overrated, underrated or reversed depending on whether ploidy dissimilarities were present in fertility, growth or looping rates (Fig. 3 a, b and c). The slopes show the sensitivity of the H:D to the ratio between ploidy dissimilar vital rates. The fertility slope was negative (Fig. 3a) because through fertility one phase contributes to the other. So, the H:D spatial pattern was reversed from the (*f_H_*:*f_D_*) spatial pattern. The growth slope was negative for the spores and smaller ramets while positive for the bigger ramets (Fig. 3b). So, the H:D spatial pattern was reversed from the *g_H_*:*g_D_* spatial pattern when estimated within the first size class and directly proportional to the *g_H_*:*g_D_* spatial pattern when estimated within the second or third size classes. The looping slope was always highly positive (Fig. 3c). So, the H:D spatial pattern was always overrated to the *l_H_*:*l_D_* spatial pattern.

### Effects of ploidy dissimilarities in fertility, growth and looping with spore dispersal

**Figure 4:**
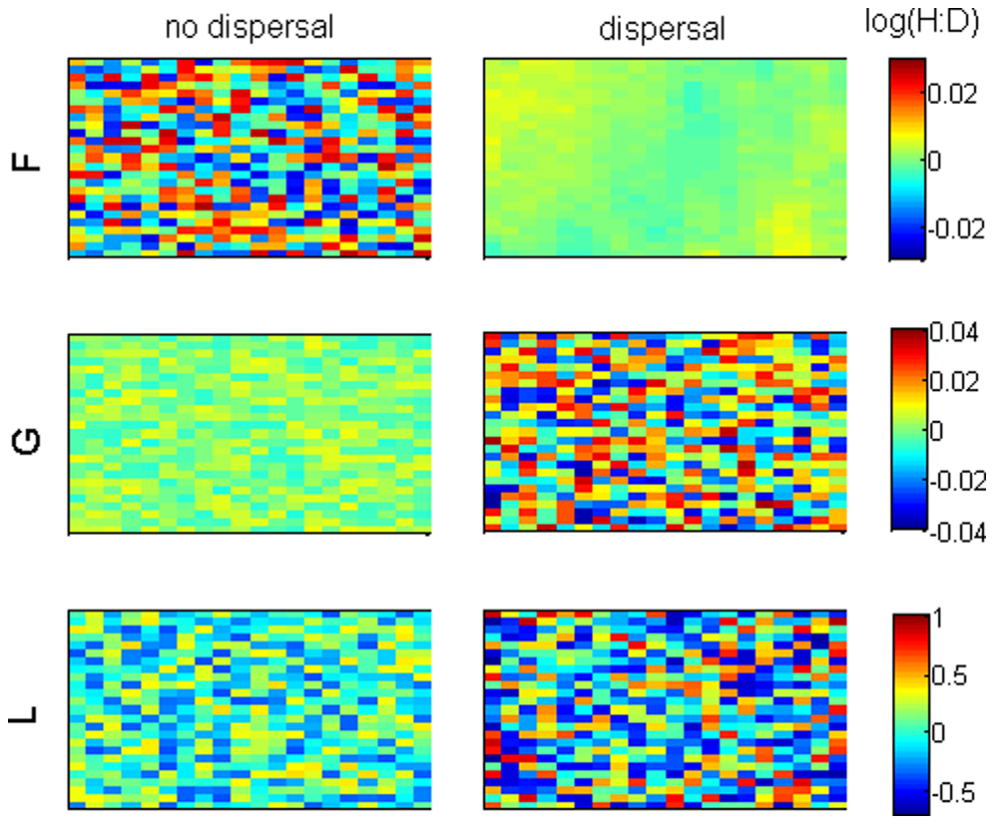
Stable H:D distribution simulated by the random model. The *disx* × *disy*  = 0.04. F: fertility life strategy with dissimilarities imposed over *f*. G: growth life strategy with dissimilarities imposed over *g*. L: looping life strategy with dissimilarities imposed over *l*. No dispersal: *dr_tet_* = *dr_carp_* = 0.1. Dispersal: *dr_tet_* = *dr_carp_* = 2.

**Figure 5:**
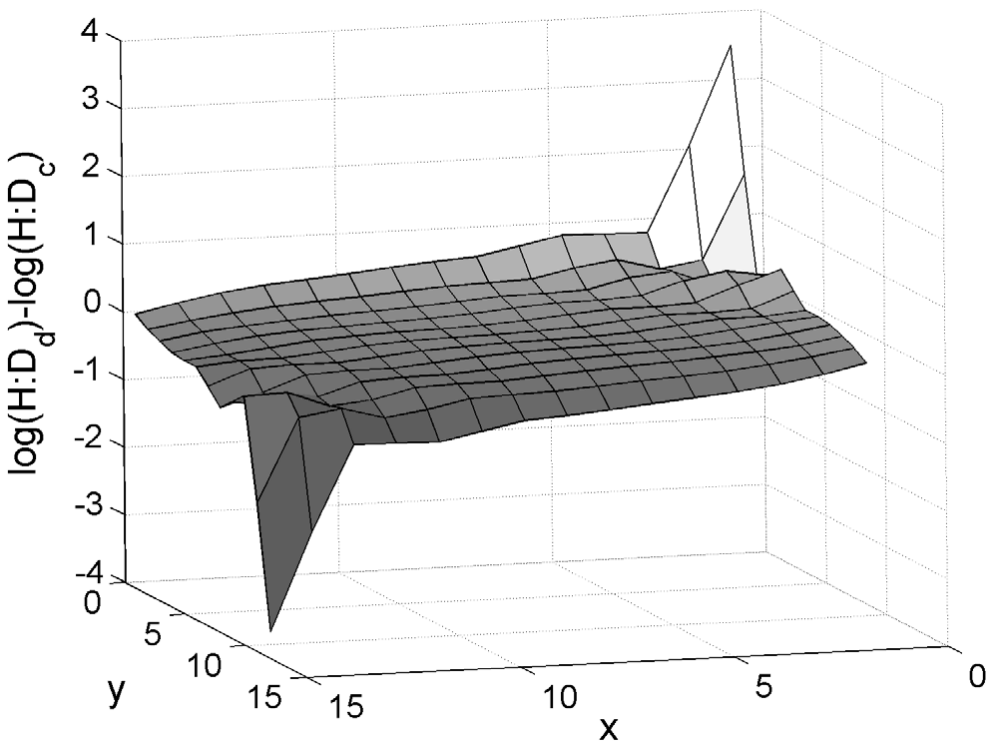
Effect of spore dispersal on the H:D. Difference between the H:D with spore dispersal (*dr_tet_* = *dr_carp_* = 2) and without spore dispersal (*dr_tet_* = *dr_carp_* = 0.2) in a looping life strategy (L) with dissimilarities imposed over looping rates (*l*) by the linear gradient model. z axis: log(H:D_dispersal_)-log(H:D_control_). *Disx*  = 0.5, *disy*  = 0.05. *f* = 5, *g* = 0.1, *l* = 0.8, *ss_carp_* = *ss_te_*_t_ = 10^−2.5^, *sw_carp_* = *sw_tet_* = 10^−2.5^ and *wf* = [0 10 500 1000 0 10 500 1000].

Spore dispersal set a connectivity smoothing the **Fv** fertility vector along the population grid. Hence, if ploidy dissimilarities were imposed over fecundity (*f*) the H:D spatial variability was smoothed relatively to the non-dispersive simulations (Fig. 4F). Spore loss through the grid borders decreased the overall fertility and the population growth rate (λ) while increasing the sensitivity of the population to the fate of the ramets (i.e. to the growth (*g*) and looping (*l*) rates in **T**). So, if ploidy dissimilarities were imposed over **T** in growth (G) or looping (L) life-strategies, the effect of spore loss overtook the effect of connectivity to enhance the H:D spatial variability (Fig. 4, G and L). This was particularly evident for dissimilarities imposed over looping rates in L life-strategies. Furthermore, as spores were lost through the edges of the population, abundances and supply of recruits was weaker there. Therefore, marginal locations were more dependent on their own demographic conditions. When these were of ploidy dissimilarities in **T** the H:D was particularly hyped (Fig. 5). Increasing spore dispersal had a stronger effect in the H:D through spore loss than through connectivity and also stronger in the marginal locations than in the bulk population.

**Figure 6:**
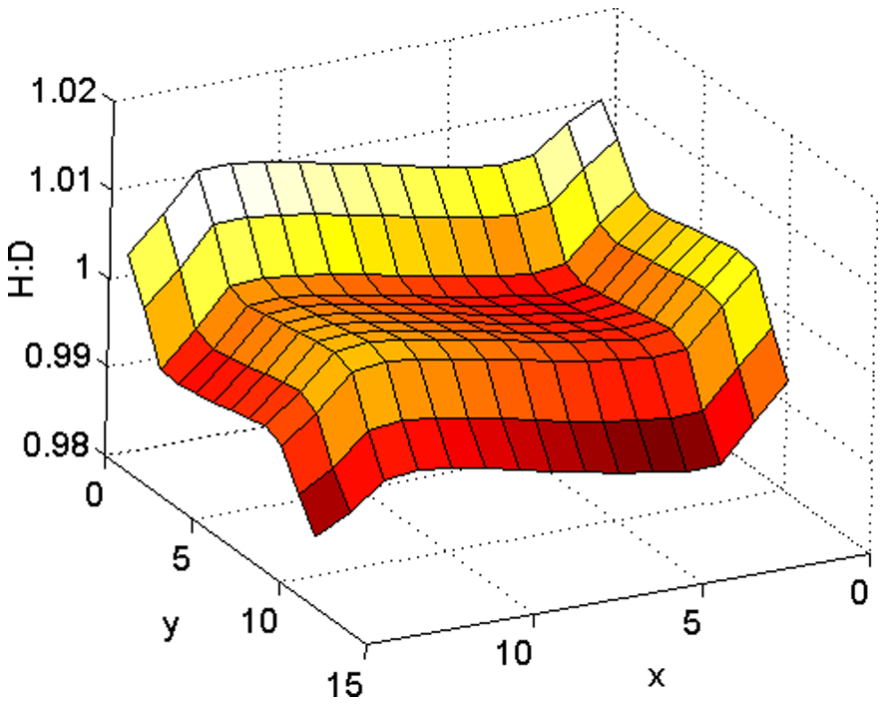
Awkward effect in the H:D spatial distribution of ploidy dissimilarities in *g*. The linear gradient model was used. Color scale: H:D. *disx*  = 0.2, *disy*  = 0.2, *dr_tet_* = *dr_carp_* = 3, *sw_tet_* = *sw_carp_* = 10^−1.5^, *ss_tet_* = *ss_carp_* = 10^−2^, *f* = 5, *wf* = [0 10 500 1000 0 10 500 1000], *g* = 0.5 and *l* = 0.1.

In the previous section addressing the absence of dispersal, it was shown that when the reproductive out-put is large the ramets' growth rates also contribute significantly to the smaller ramets of the opposite phase. Thus, the H:D spatial distribution missfitted the *g_H_*:*g_D_* spatial distribution. When spore dispersal was set the diverging effects of connectivity and spore loss were added resulting in awkward and almost unpredictable H:D spatial patterns (like in Fig. 6) completely miss-fitting the fitness ratio given by the *g_H_*:*g_D_* (in Fig. 2 upper panel).

Equation 5 was applied to the set of accepted demographic matrices being observed that at steady-state as λ tended to be predominantly elastic to the fate of the ramets (in **T**) the asymptotic population structure at any point (**Nx,y**) was set by the ploidy dissimilarities in the fate of the ramets. On the other hand, as λ tended to be predominantly elastic to fertility (in **Fv**) the asymptotic population structure at any point was set by the ploidy dissimilarities in fertility. Therefore, in real world situations, despite spatial heterogeny and dispersion the driver for an uneven H:D must be common to an entire population.

### Effects of ploidy dissimilar spore dispersal

**Figure 7:**
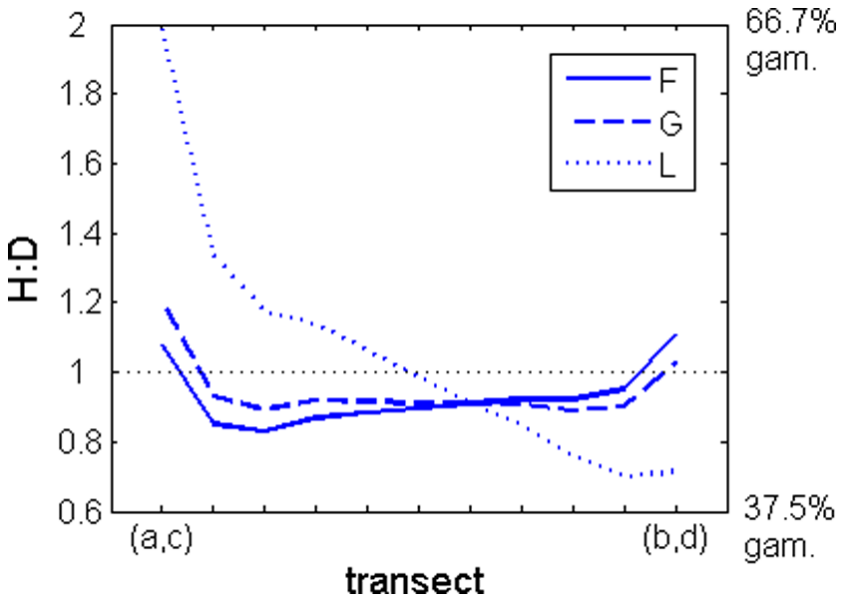
Stable H:D along a transect from (*a*,*c*) to (*b*,*d*). Simulations of populations with fertility (F), growth (G) and looping (L) life strategies with ploidy dissimilarities in the respective *f*, *g* and *l* rates and in *dr*. Gametophytes (gam) are the haploids.

The phase dispersing less dominated the bulk of the population by dominating local recruitment, but was dominated in the marginal locations as these are more dependent on immigrant spores. When dissimilarities in fertility, growth or looping were combined, the H:D asymptotic distribution was the linear combination of the distributions imposed by both dissimilarities (in fertility, growth or looping and in dispersal: Fig. 7). However, the linear combinations were not equally weighted. For the simulations over fertility or growth life strategies the effect of the ploidy dissimilarities in fertility or growth rates was veiled by the effect of ploidy dissimilarities in spore dispersal (Fig. 7 lines F and G), whereas for the simulations over looping life strategies the effect of the ploidy dissimilarities in looping rates largely overcame the effect of the ploidy dissimilarities in spore dispersal (Fig. 7 line L). In this L simulation *disx* and *disy* were half of what they were in the F and G simulations and the maximum difference between *l_H_* and *l_D_* was one tenth of the difference between *dr_carp_* and *dr_tet_*.

### Transient dynamics

The transient properties of the H:D dynamics were better unveiled testing the “linear gradient” dissimilarity hypothesis. Results are shown in animations of the initial 60 month periods during which the color scale representing the H:D is adjusted at particular times. It was imposed in the same spatial pattern as in figure 1 where haploid vital rates dominated in lower-left corner whereas diploid vital rates dominated the upper-right corner. In the absence of spore dispersal following colonization patches of ploidy dominance were formed. In populations with fertility life strategies and ploidy dissimilarities over fertility rates (F) these patches were dominated by the less fertile (and thus less fit) ploidy and oscillated until settlement in the stable H:D (Animation A in File S1). For populations with growth life strategies and ploidy dissimilarities over growth rates (G) the patches suffered conspicuous changes in ploidy dominance (Animation A in File S2) implying cyclic dominance of the less fit phase. In populations with looping life strategies with ploidy dissimilarities over looping rates (L) the patches were always dominated by the fitter ploidy and tended slowly and monotonically to the stable H:D (Animation A in File S3). Introducing spore dispersal smoothed the formation of patches and its oscillation in F simulations (Animation B in File S1), enhanced it in L simulations (Animation B in File S3) and turned them irregular in G simulations (Animation B in File S2), as a consequence of the peculiar role of growth on ploidy phase dominance previously presented. The effect of ploidy dissimilar spore dispersal was tested with colonization starting in the population centre. Then, were formed patches dominated by the first ploidy to arrive. These patches quickly oscillated in F and G simulations (Animations C in File S1 and Animation C in File S2) and slowly evolved in L simulations (Animation C in File S3), but always tending to a situation where the less dispersive ploidy was favored in the center of the population whereas the more dispersive ploidy was favored in its edges. The ploidy dissimilarities in spore dispersal conjugated evenly with the ploidy dissimilarities in fecundity (Animation D in File S1). Furthermore, the oscillations increased in amplitude and duration, revealing an interaction between both. The effects of ploidy dissimilarities in spore dispersal overwhelmed the effects of ploidy dissimilar growth (Animation D in File S2) but were offset by the effects of ploidy dissimilarities in looping on the long run (Animation D in File S3). There was a huge interaction between ploidy dissimilarities in looping and in spore dispersal extending both the duration of the transient phase and the heterogeny of the H:D at steady-state (i.e. the sensitivity of the ploidy dominance to the ploidy dissimilarities in looping rates).

### Gelidium sesquipedale

**Figure 8:**
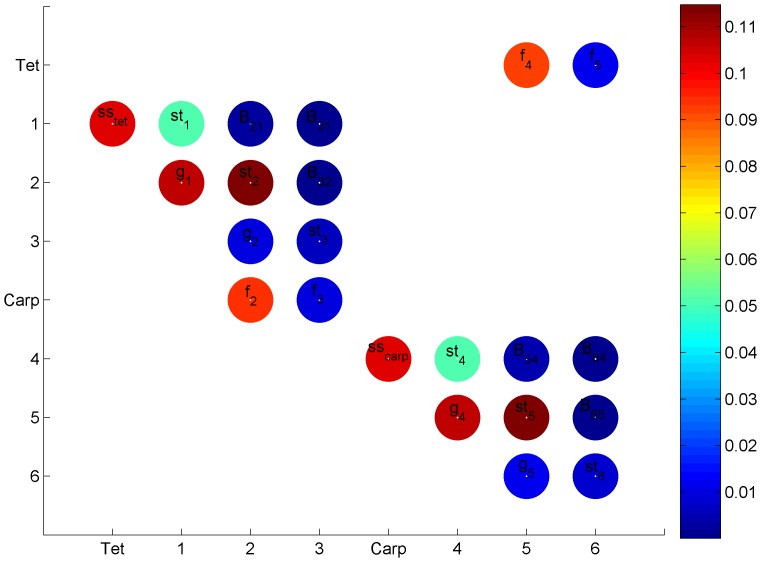
Elasticities of λ to the *Gelidium sesquipedale*'s vital rates. The demographic model was built from previous data [1], [9], [10].

The spore survival and dispersion rates were unknown and thus fit to yield realistic population growth rates (λ). In the absence of dispersal, with *sw_tet_* = *sw_carp_* = 10^−1.8^ and *ss_tet_* = *ss_carp_* = 10^−2^ each location grew at λ = 1.028. The overall spore survival of 10^−3.8^ is well within the range reported in the literature. The transient phase was short with oscillations quickly damping away. It took only 10 months for the change in λ to be always <1% of its current value and 15 months to be always <0.1%. In the absence of spore dispersal each fertility component (in equation 2) could be allocated to its proper demographic matrix entry (in equation 6) with the elasticities of λ following the principles by Caswell [30], Franco and Silvertown [29] and Oostermeijer *et al.* [25]. The sum of the elasticities of λ to all fertility rates were 0.206, to all spore survival rates while settled were 0.206, to all growth rates were 0.235 and to all looping rates were 0.353. Both λ and the HD were mainly elastic to vital rates in the shortest loop within the life cycle (Fig.8). This loop was the sequence of spores surviving and germinating to become small ramets (*ss_tet_* and *ss_carp_*), growing from small to medium sized ramets (*g_1_* and *g_4_*), reproducing once medium sized (*f_2_* and *f_5_*) and surviving as so (*st_2_* and *st_4_*), thus extending reproduction. The higher rates of carpospore production and tetrasporophyte growth resulted in an HD of 0.736, corresponding to 57.6% dominance of the diploids irrespective of space location. This was far from the 80% diploid dominance reported by Carmona and Santos [10]. Introducing the dissimilarities in spore germination along the spatial gradient caused the ploidy dominance to shift from 77.5% to 43% diploids, but only at very limited (both in space and time) situations during the transient oscillations (Animation S4). Eventual ploidy dissimilarities in looping rates did not exchange the scenario. 50% increases in any of the ploidies' looping were tested and found unable to produce consistent 80% diploid abundances or patches of haploid dominance. This was only possible conjugating the observed ploidy dissimilarities over ramet growth and fertility with hypothetical ploidy dissimilarities over spore dispersal. In the example shown in Animation S5 increasing Carpospore dispersal to twice that of Tetraspores yielded consistent patches of overwhelming ploidy dominance.

## Discussion

The introduction of spore dispersal and settlement in a fine scale spatially heterogenic environment approximated the simulations from real world situations relative to previous non-dimensional modeling. It was revealed that a fine scale spatial heterogeneity (i.e. at the intra-population level) conjugated with ploidy dissimilar vital rates (i.e. conditional differentiation) has a high potential to set the niche partitioning Hughes and Otto [8] argued necessary for the prevalence of isomorphic biphasic life cycles. However, for this to take place one of two conditions must be met: (1) when the ramets of both ploidies differentiate their survival responses to a changing environment or (2) when the conditional differentiation of both ploidies in some vital rates are conjugated with tetraspores and carpospores exhibiting largely different dispersive capabilities.

Evidence for ploidy dissimilar fecundity rates has already been discussed in several studies [11], [14], [22], [31], [32], but without reporting any fine scale spatial variability. This work showed the H:D spatial distribution would always be reversed relative to the spatial distribution of ploidy differences in fecundity. Therefore, at each location the population would always be dominated by the phase locally less fit, i.e. with smaller fecundities. Furthermore, the dominant effect of spore dispersal would be setting a connectivity smoothing the H:D spatial pattern. These results contradict the ones from another theoretical modeling by Fierst et al [23] stating biphasic life cycles exhibit uneven H:D as a consequence of ploidy dissimilar fertility rates. Thornber and Gaines [22] found no correlation between the spatial distribution of the ploidy fertilities ratio and the ploidy abundances ratio of *Mazzaella flaccida*. They proposed fixed ploidy dissimilar fertility rates as the cause for the global average H:D but not for its spatial variability.

Evidence for ploidy dissimilar ramet growth rates were reported by Destombe *et al*. [12], Gonzalez and Meneses [13], Garza-Sanchez *et al.* [14], Carmona and Santos [10] and Pacheco-Ruiz *et al.* [16]. However, these studies also fail to report any fine scale spatial variability. This work shows that the resulting H:D spatial distribution can easily mismatch the spatial distribution of the ploidy differences in ramet growth. The reason for this occurrence is the phase growing better contributes to its own dominance over the bigger ramets but also to the dominance of the opposite phase over the smaller ramets, as already demonstrated by Vieira and Santos [24]. Thus, the spatial patterns of ploidy dominance became very smooth, atypical and totally miss-fitting the ploidy fitness ratio. It was even possible to find the local dominance of the phase less fit, i.e. with smaller growth rates, particularly following colonization. Therefore, despite the existence of ploidy dissimilar growth rates, these alone are unlikely to generate an advantageous niche partition.

A number of studies present evidences for ploidy dissimilar looping rates [10], [12], [13], [15], [16]. This work has shown the spatial distribution of the H:D always matches the spatial distribution of the ploidy differences in ramet looping and even supersedes it. This is true for any location within the population, following colonization or at the asymptotic stable population structure. Therefore, a conditional differentiation of the ramets leading to ploidy dissimilar looping rates are an excellent means for the niche partition required for the stability and evolution of biphasic life-cycles [8] and observed at a spatially fine scale [2], [4], [6], [7], [12], [20]. It is particularly interesting the shift of phase dominance at a very short spatial scale (few meters) for populations of *Gelidium canarensis* in the stable population structure [20]. The present work suggests such shifts can hardly be caused by anything else besides ploidy dissimilar looping rates in populations with survival life strategies. Barbuti *et al*. [33] studied the role of sexual reproduction (fertility) *vs* asexual reproduction (a looping rate) in the population structure and dynamics of *Carassius gibelio*, a cyprinid fish with a life cycle entirely different from the isomorphic biphasic of some seaweeds. These authors also found the looping rate to be of utmost importance by amplifying the locally fitter genotypes. Contrary to the results by previous non-spatial modeling attempts, ploidy differences in looping rates need not be balanced by ploidy differences in some other demographic trait: they are self-balanced by their fine scale spatial variability. Furthermore, there is a synergistic effect between eventual ploidy dissimilarities in looping rates and the fact that spores disperse: with a reasonable amount of spores inevitably lost from the population through its edges the peripheral locations become particularly sensitive to the fate of the ramets and their ploidy dissimilarities. Then, niche partition becomes particularly evident and effective in these marginal locations. In a study about the spatial genetic structure of *Thuja occidentalis*, a conifer with a life cycle entirely different from the isomorphic biphasic of some seaweeds, Panday and Rajora [34] also determined that peripheral populations were much more heterogenic than the bulk populations.

In the current model the spore dispersal was simulated by giving it a maximum dispersive range over the projection interval of one month. With a finer temporal and biological resolution this can be traced back to the amount of time spores take to settle and their mortality rates while suspended. Such spore performance has often been reported to be ploidy dissimilar [10], [12], [13], [14], [29], [35] and its effect on an uneven H:D was experimentally demonstrated by Pacheco-Ruiz *et al*. [16]. The current work has demonstrated ploidy dissimilarities in the spores' ability to disperse can also create the required niche partition even in spatially homogeneous environments. Then, in populations with looping life strategies the H:D patches formed show great constancy, whereas in populations with fertility or growth life strategies the patches may oscillate backward and forward at a fast rate. A similar transient behaviour was obtained in Vieira and Santos [18] for the non-spatial case.

The *Gelidium sesquipedale*'s estimated demography corresponds to those predicted by Vieira and Santos [18], [24] being approximately evenly elastic to fertility, growth and looping rates. In these, individuals flow mainly through the shortest life-cycle loop generating short period oscillations on both λ and the H:D quickly damping away. Demographic properties like λ or the HD are little elastic to any vital rates thus requiring huge ploidy dissimilarities, even in looping rates, to yield conspicuous ploidy field dominances. Therefore it was not surprisingly that the simulations of the ploidy dissimilarities estimated by Carmona and Santos [10] were unable to consistently generate the ploidy field dominance observed by these same authors or a spatial niche partition. Ranges of spore dispersal are unknown and their simulation may be a wild guess. Nevertheless, given the cytological differences between tetraspores and carpospores it is reasonable to expect them having largely different dispersive abilities. Such simulations generated patches that although transient exhibited enormous dominance (sometimes close to 100%) for either ploidy over wide areas and long periods. Most often diploids dominated often overcoming the 80% dominance observed by Carmona and Santos [10]. However, they also generated patches of transient field dominance by the ploidy locally less fit casting doubts about the efficiency of conjugating ploidy uneven spore dispersal with conditional differentiation of the ramets or germinating spores.

## Supporting Information

Appendix S1 **Analytical solution of the local stable population structure.**
(DOC)

Animation S1 **H:D transient trajectory for a population with a F life strategy.** Ploidy dissimilarities in the f rates according to the linear gradient hypothesis. Ploidy even colonization (**A**, **B**) along the entire population or (**C**, **D**) on its centre. The colour scale is *log*(H:D) and it changes bounds for *t*>13. (**A**) with ploidy dissimilarities in *f* and without spore dispersal. *dr_carp_* = *dr_tet_* = 0.5, *disx* = *disy* = 0.5, *f* = 10; (**B**) with ploidy dissimilarities in *f* and with spore dispersal. *dr_carp_* = *dr_tet_* = 5, *disx* = *disy* = 0.5, *f* = 15; (**C**) with ploidy dissimilarities only in spore dispersal. *dr_carp_* = 3, *dr_tet_* = 6, *disx* = *disy* = 0 and *f* = 15; (**D**) with ploidy dissimilarities in *f* and in spore dispersal. *dr_carp_* = 3, *dr_tet_* = 6, *disx* = *disy* = 0.5 and *f* = 10; (**all**) *ss_tet_* = *ss_carp_* = 10^−1.8^, *sw_tet_* = *sw_carp_* = 10^−1.2^, *g* = 0.12, *l* = 0.05, **wf** = [0 10 500 1000 0 10 500 1000], *a* = 1, *b* = 15, *c* = 1, *d* = 15 and Δ*x* = Δ*y* = 1.
(AVI)

Animation S2 **H:D transient trajectory for a population with a G life strategy.** Ploidy dissimilarities in the *g* rates according to the linear gradient hypothesis. Ploidy even colonization (**A**, **B**) along the entire population or (**C**, **D**) on its centre. The colour scale is *log*(H:D) and it changes bounds for *t*>20 and *t*>40. (**A**) with ploidy dissimilarities in *g* and without spore dispersal. *dr_carp_* = *dr_tet_* = 0.5, *disx* = *disy* = 0.2, *f* = 3; (**B**) with ploidy dissimilarities in *g* and with spore dispersal. *dr_carp_* = *dr_tet_* = 3, *disx* = *disy* = 0.2, *f* = 4; (**C**) with ploidy dissimilarities only in spore dispersal. *dr_carp_* = 3, *dr_tet_* = 6, *disx* = *disy* = 0, *f* = 5; (**D**) with ploidy dissimilarities in *g* and in spore dispersal. *dr_carp_* = 3, *dr_tet_* = 6, *disx* = *disy* = 0.2, *f* = 5; (**all**) *ss_tet_* = *ss_carp_* = 10^−2^, *sw_tet_* = *sw_carp_* = 10^−1.5^, *g = *0.6, *l* = 0.05, **wf** = [0 10 500 1000 0 10 500 1000], *a* = 1, *b* = 15, *c* = 1, *d* = 15 and Δ*x* = Δ*y* = 1.
(AVI)

Animation S3 **H:D transient trajectory for a population with a L life strategy.** Ploidy dissimilarities in the *l* rates according to the linear gradient hypothesis. Ploidy even colonization (**A**, **B**) along the entire population or (**C**, **D**) on its centre. The colour scale is *log*(H:D) and it changes bounds for *t*>20 and *t*>50. (**A**) with ploidy dissimilarities in *l* and without spore dispersal. *dr_carp_* = *dr_tet_* = 0.5, *disx* = *disy* = 0.1, *f* = 3;.(**B**) with ploidy dissimilarities in *l* and with spore dispersal. *dr_carp_* = *dr_tet_* = 5, *disx* = *disy* = 0.1, *f* = 3; (**C**) with ploidy dissimilarities only in spore dispersal. *dr_carp_* = 3, *dr_tet_* = 6, *disx* = *disy* = 0, *f* = 6; (**D**) with ploidy dissimilarities in *l* and in spore dispersal. *dr_carp_* = 3, *dr_tet_* = 6, *disx* = *disy* = 0.1, *f* = 5; (**all**) *ss_tet_* = *ss_carp_* = 10^−1.8^, *sw_tet_* = *sw_carp_* = 10^−1.2^, *g* = 0.02, *l* = 0.8, **wf** = [0 10 500 1000 0 10 500 1000], *a* = 1, *b* = 15, *c* = 1, *d* = 15 and Δ*x* = Δ*y* = 1.
(AVI)

Animation S4 **Predicted transient ploidy dominance for** ***G. sesquipedale*** **.** Simulation over a 4 year period starting with ploidy even colonization in 7 random points along the population. Ploidy dissimilarities in spore attachment simulated according to the linear gradient hypothesis with disx  = 1 and disy  = 0. *dr_carp_* = *dr_tet_* = 2, *f* = 7000, **wf** = [0 0 2 4 0 0 1 2], *ss_tet_* = *ss_carp_* = 10^−2^, *sw_tet_* = *sw_carp_* = 10^−1.5^, Matrix T entries given by equation 6. *a* = 1, *b* = 20, *c* = 1, *d* = 20 and Δ*x* = Δ*y* = 1.
(AVI)

Animation S5 **Predicted transient ploidy dominance for** ***G. sesquipedale*** **with hypothetical ploidy uneven spore dispersal.** Simulation over a 4 year period starting with ploidy even colonization in 7 random points along the population. Ploidy dissimilarities in spore attachment simulated according to the linear gradient hypothesis with disx  = 1 and disy  = 0. *dr_carp_* = 4, *dr_tet_* = 2, *f* = 7000, **wf** = [0 0 2 4 0 0 1 2], *ss_tet_* = *ss_carp_* = 10^−2^, *sw_tet_* = *sw_carp_* = 10^−1.5^, Matrix T entries given by equation 6. *a* = 1, *b* = 20, *c* = 1, *d* = 20 and Δ*x* = Δ*y* = 1.
(AVI)
