# Supplementary material for: Regulation of the Demographic Structure in Isomorphic Biphasic Life Cycles at the Spatial Fine Scale
Source: PLoS One. 2014 Mar 21;9(3):e92602. doi: 10.1371/journal.pone.0092602 (PMC3962440; doi:10.1371/journal.pone.0092602)
Supplement: Appendix S1 — Analytical solution of the local stable population structure. (DOC) [file pone.0092602.s001.doc]

**Appendix S1 - analytical solution of the local stable population structure**.

The deduction for any point (x,y) started by assuming as the new starting time (t_0_) one where the whole population was already at its stable structure and asymptotic growth rate (λ). Then, the fertility vector had the solution:

$F_{\left(x_{0},y_{0},t+t_{0}\right)}=\lambda^{t}.F_{\left(x_{0},y_{0},t_{0}\right)}=\lambda^{t}\left[\begin{matrix} tet_{\left(x_{0},y_{0},t_{0}+1\right)} \\ 0 \\ 0 \\ 0 \\ carp_{\left(x_{0},y_{0},t_{0}+1\right)} \\ 0 \\ 0 \\ 0 \end{matrix}\right]$ (1)

where λ was estimated as (N_t+t0_/N_t0_)^1/t^. Since the fertility vector Fv could be written as a function of its initial structure, it was possible to estimate N_t+t0_ iterating equation (5) in the bulk manuscript from N_t0_. Neglecting the x_0_ and y_0_ subscripts for space location and standardizing the population to its initial size:

$\begin{matrix} \frac{N_{1+t_{0}}}{\lambda}=\frac{TN_{t_{0}}+Fv_{t_{0}}}{\lambda}=TN_{t_{0}}\lambda^{-1}+Fv_{t_{0}}\lambda^{-1} \\ \frac{N_{2+t_{0}}}{\lambda^{2}}=\frac{T\left(TN_{t_{0}}+Fv_{t_{0}}\right)+F_{t_{0}}\lambda}{\lambda^{2}}=T^{2}N_{t_{0}}.\lambda^{-2}+TFv_{t_{0}}\lambda^{-2}+Fv_{t_{0}}.\lambda^{-1} \\ \frac{N_{t+t_{0}}}{\lambda^{t}}=\underbrace{T^{t}N_{t_{0}}\lambda^{-t}}_{A}+\underbrace{\sum_{i=1}^{t} \left(T^{i-1}\lambda^{-i}\right)Fv_{t_{0}}}_{B} \end{matrix}$ (2)

The term A was relative to the fate of the individuals already in the population at t_0_ while the term B was relative to the production and fate of new individuals. The sum in B is a geometric succession in matrix space with an analytical solution similar the one‑dimensional case. This is possible provided the matrix is square, non‑singular (and therefore invertible) and the unidimensional operands are replaced by the matrix operands. The solution to the population vector became:

$\frac{N_{t+t_{0}}}{\lambda^{t}}=\underbrace{T^{t}N_{t_{0}}\lambda^{-t}}_{A}+\underbrace{\lambda^{-1}inv\left(I-T\lambda^{-1}\right)Fv_{t_{0}}}_{B}$ (3)

where I is the identity matrix. Solving equation (3) when t tends to infinity, the term A tends to 0: being T a matrix only describing the fate of individuals alive, if any individual can die, eventually all will die. So the stable population structure in point (x_0_,y_0_) was given by the B term (equation 4). It was not a fully analytical solution because Fv_t0_ still had to be numerically determined.

$N_{\left(x_{0},y_{0},t_{0}\right)}=\lim_{t\to+\infty} \frac{N_{\left(x_{0},y_{0},t+t_{0}\right)}}{\lambda^{t}}=\lambda^{-1}inv\left(I-T_{\left(x_{0},y_{0}\right)}\lambda^{-1}\right)Fv_{\left(x_{0},y_{0},t_{0}\right)}$ (4)
